# Effect of Intravenous Dexmedetomidine on Spinal Anesthesia

**DOI:** 10.7759/cureus.15708

**Published:** 2021-06-17

**Authors:** Ezhil Bharthi Sekar, Usha Vijayaraghavan, A Mohammed Sadiqbasha

**Affiliations:** 1 Anaesthesiology, ACS Medical College Hospital, Chennai, IND

**Keywords:** intravenous dexmedetomidine, intrathecal bupivacaine, spinal anesthesia, post-operative analgesia, sensory and motor blockade

## Abstract

Background

Subarachnoid block (SAB) with hyperbaric bupivacaine is routinely administered for abdominal and lower limb surgeries. Various agents have been used intrathecally as adjuvants to local anesthetic to increase efficacy and prolong the duration of SAB, among which opioids and α2 agonists are most commonly used. Intravenously administered dexmedetomidine has also been shown to prolong the duration of sensory and motor blockade obtained with subarachnoid block.

Methods

A total of 212 adult patients scheduled to undergo abdominal or lower limb surgeries were enrolled in this prospective, double-blind, randomized study. They were divided into two equal groups to receive either intravenous dexmedetomidine (group D) 1μg/kg loading followed by maintenance at 0.5μg/kg or intravenous normal saline (group C) after 15 mins of subarachnoid block; no other intraoperative sedatives were used. The onset of sensory block and motor block, and the highest level of sensory block attained were assessed. The time taken for sensory regression to L1, Modified Bromage scale 0, and rescue analgesia requirement were assessed.

Results

Patients receiving intravenous dexmedetomidine showed no significant change in terms of onset and level of sensory block (P-0.774) and the onset of motor block (P-0.738). The time taken for sensory regression to L1 was significantly prolonged (P-0.000). Also, the time taken to achieve Modified Bromage scale of 0 and time taken for rescue analgesia was significantly prolonged (P-0.000).

Conclusion

Intravenous dexmedetomidine prolonged the duration of sensory and motor block, and also appears to provide sedation with easy arousability and analgesia postoperatively while maintaining hemodynamic stability with no significant side effects.

## Introduction

Central neuraxial blocks are routinely administered for abdominal and lower limb surgeries. Subarachnoid block (SAB) with hyperbaric bupivacaine is preferred over epidural anesthesia as it has a faster onset and denser block. It is cost-effective and easy to administer. Various agents have been used intrathecally as adjuvants to local anesthetic to increase efficacy and prolong the duration of subarachnoid block, among which opioids and α2 agonists are most commonly used.

Dexmedetomidine is a potent α2 agonist and has high α2 selectivity. These receptors are found in many sites throughout the body including the central nervous system (CNS), spinal, and peripheral tissues. The receptors in locus coeruleus are responsible for sedation, analgesia, anxiolysis, and sympatholysis. At the spinal cord, stimulation of α2 receptors at the substantia gelatinosa of the dorsal horn leads to inhibition of the release of substance P. The spinal mechanism is principal for the analgesic effects of dexmedetomidine even though there is evidence for both supraspinal and peripheral sites of action.

Dexmedetomidine has also been shown to prolong the duration of sensory and motor blockade obtained with SAB while maintaining patient arousability. Various studies have administered intravenous dexmedetomidine at 1 mcg/kg loading dose over 10-20 mins and maintenance dose of 0.4-0.5 mcg/kg/hr, and found that the sensory and motor blocks of spinal anesthesia were prolonged with good sedation and few side effects when used as an adjuvant to prilocaine, hyperbaric ropivacaine, isobaric and hyperbaric bupivacaine [1-4]. It did not cause any impairment or disinhibition of cognitive function.

We decided to conduct a study with the primary aim of evaluating the efficacy of intravenously administered dexmedetomidine on the onset, duration, and regression of hyperbaric bupivacaine-induced spinal anesthesia. The secondary outcomes in our study were to evaluate the effects on hemodynamic parameters, adverse effects if any, and sedation.

## Materials and methods

This randomized prospective comparative double-blind study was conducted in ACS Medical College Hospital, India. from June 2019 to March 2021, after approval from Institutional Ethical Committee, and written informed consent was obtained from all patients participating in the study before surgery. Keeping the power at 80% and confidence interval at 95% to detect at least a difference of 10±22.4 mins in meantime to sensory regression to L1 between the groups, the minimum sample size required was calculated to be 158 patients. However, we included 212 patients for better validation of results.

The study was conducted on 212 adult patients of ASA grade I or II, between 18-60 years of age, of either sex, undergoing lower abdominal and lower limb surgeries. Exclusion criteria included patient refusal, ASA III and IV class of patients, patients on any opioid or sedative medication, history of alcohol or drug abuse, known allergy to any of the test drugs, contraindication to spinal anesthesia, patients on calcium channel blockers /angiotensin-converting enzyme (ACE) inhibitors/clonidine/β-blockers use.

The patients were randomized into two groups of 106 patients each using sequentially numbered envelopes. To maintain the double-blind nature of the study, the randomization and preparation of drugs for injection were done by an anesthesiologist not involved in the study. The anesthesiologist who gave the study drugs and recorded data were also blind to the patient group assignment. Group D received intravenous dexmedetomidine 1μg/kg as a loading dose over 10 mins, followed by a maintenance dose of 0.5μg/kg/hr by infusion pump till 10 mins before the end of surgery. Group C received intravenous normal saline in equivalent loading and maintenance doses by infusion pump till 10 mins before the end of surgery.

All patients were fasted overnight with clear fluids allowed until 4 hours preoperatively and none of them received any sedative premedication. On arrival to the operation theatre, ECG, heart rate, respiratory rate, SpO2, and NIBP were monitored every 5 minutes, all patients were preloaded with Ringer Lactate solution 10ml/kg. SAB was performed in L3-L4 space with 25G Quincke's needle by midline approach using 3.5cc vol of 0.5% heavy bupivacaine. Oxygen was administered via a mask at 2-4l/min. After 15 mins of SAB, the prepared drug was started as an intravenous infusion. Loading dose was given over 10 mins and maintenance dose started thereafter until 10 mins before the end of surgery. Both the groups received no other intraoperative sedatives. 

Pulse rate (PR), blood pressure (BP), oxygen saturation (SpO2), respiratory rate (RR), and motor block was evaluated using modified Bromage scale (grade 0: no paralysis, grade 1: unable to move hip, grade 2: unable to move hip and knee, grade 3: unable to move hip, knee, and ankle); sensory block was assessed using pinprick method and level of sedation using Ramsay Sedation Scale(RSS) Score at intervals of 1, 3, 5 and every 5 mins until 20 mins and every 10 mins thereafter until 300 mins were noted.

Any side effects developed by the patient were noted and treated. Bradycardia was defined as HR≤50/min and was treated with intravenous glycopyrrolate 0.2mg. Hypotension was defined as fall in SBP > 30% of pre-op values/ <90mmHg and was treated with 200ml boluses of crystalloids, and if persisted, then injection ephedrine was used. Respiratory depression was defined as RR <9/min or SpO2<90%. Excessive sedation was defined as RSS Score greater than 4. Patients were shifted to the recovery room at the end of surgery and shifted to ward after sensory regression to L1 and modified Bromage scale 0. In patients who complained of pain postoperatively, 1g IV paracetamol was given as rescue analgesia.

The following parameters were recorded: time taken for the highest level of sensory blockade, time taken for the motor blockade to reach Modified Bromage scale 3, time taken for sensory regression to L1 level, regression of motor blockade to Modified Bromage scale 0, and time for first rescue analgesia requirement postoperatively.

Data were analyzed using a statistical package for social sciences (SPSS), Continuous variables were expressed as mean and standard deviation and analyzed using the Student t-test. For RSS Score, the Mann-Whitney test was used to analyze the medians from the two study groups. A P < 0.05 was considered statistically significant. 

## Results

All the patients in groups C and D were comparable demographically (age, height, weight, sex) (Table 1).

**Table 1:** Demography of Patients

Parameters	Group C	Group D	P-value (2 Sample t test)
No. of Patients	106	106	-
Age in years (Mean ± S.D.)	35.83 ± 12.60	35.3 ± 9.64	0.855 (ns)
Height in cm. (Mean ± S.D.)	161.07 ± 6.97	163 ± 6.12	0.258 (ns)
Weight in kg. (Mean ± S.D.)	58.43 ± 8.65	63.5 ± 10.16	0.42 (ns)
Sex(M/F)	17/13	17/13	-
Values are Mean ± S.D, P<0.05 - statistically significant*, P value >0.05 – statistically not significant (ns)			

**Figure 1:**
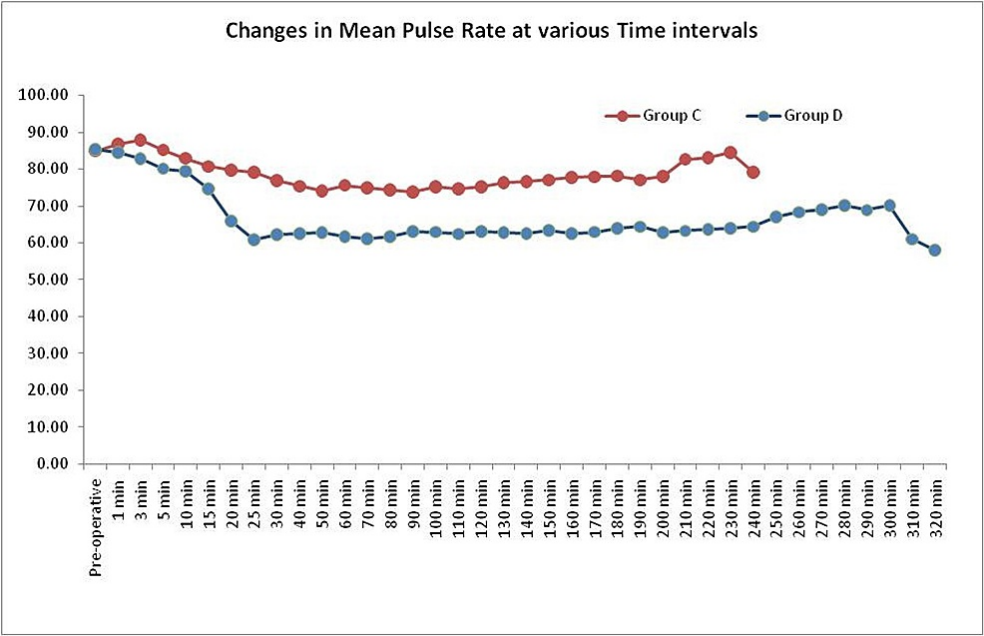
MEANS OF PULSE RATE IN BOTH GROUPS

The baseline HR was similar in both groups. The PR in group C remained relatively stable around the baseline and no patients developed bradycardia. In group D the PR started falling from 85.37 to 60.87 at 25th minute, following this the PR remained around that level. The maximum fall in PR was noted in group D (28.70% fall from the baseline at 25th minute). The PR showed significant difference between both the groups from 15 minutes. Bradycardia < 60 was seen in 50% patients in group D of which 10 patients showed HR≤ 50 requiring injection glycopyrrolate (Table 2) (Figure 1).

**Table 2:** Comparison of Changes in Pulse Rate

Time	Group C (Mean ± S.D.)	Group D (Mean ± S.D.)	P-Value (2 Sample t-Test)
Pre-operative	(84.87±13.11)	(85.37±12.11 )	0.879 (ns)
1min	(86.73±13.38) (2.20)	(84.50±12.40) (-1.02)	0.505 (ns)
3min	(87.87±13.24) (3.53)	(82.73±13.49) (-3.09)	0.142 (ns)
5min	(85.17±2.70) (0.35)	(80.10±13.01) (-6.17)	0.132 (ns)
10min	(82.87±12.32) (-2.36)	(79.37±12.86) (-7.03)	0.266 (ns)
15min	(80.67±11.63) (-4.95)	(74.57±9.29) (-12.65)	0.029*
20min	(79.73±11.31) (-6.05)	(66±9.09) (-22.69)	0.000*
25min	(79±10.77) (-6.92)	(60.87±6.80) (-28.70 )	0.000*
30min	(76.87±9.71) (-9.43)	(62.30±6.34) (-27.02)	0.000*
40min	(75.27±8.92) (-11.32)	(62.50±6.50) (-26.79)	0.000*
50min	(73.97±9.05) (-12.85)	(62.90±6.55) (-26.32)	0.000*
60min	(75.53±9.98) (-11.00)	(61.63±6.67) (-27.80)	0.000*
70min	(74.83±9.90) (-11.83)	(61.07±6.84) (-28.47)	0.000*
80min	(74.30±9.69) (-12.45)	(61.63±6.55) (-27.80)	0.000*
90min	(73.70±9.76) (-13.16)	(63.07±9.31) (-26.13)	0.000*
100min	(75.17±10.28) (-11.43)	(62.93±9.64) (-26.28)	0.000*
110min	(74.50±9.97) (-12.22)	(62.40±9.67) (-26.91)	0.000*
120min	(75.13±10.37) (-11.47)	(63.10±9.94) (-26.09)	0.000*
130min	(76.27±12.88) (-10.47)	(62.83±8.52) (-26.40)	0.000*
140min	(76.63±12.97) (-9,71)	(62.53±7.49) (-26.75)	0.000*
150min	(76.97±13.37) (-9.31)	(63.40±6.99) (-25.74)	0.000*
160min	(77.63±13.69) (-8.53)	(62.53±6.74) (-26.75)	0.000*
170min	(77.83±13.02) (-8.29)	(62.93±6.33) (-26.28)	0.000*
180min	(78.07±11.50) (-8.02)	(64±8.07) (-25.03)	0.000*
190min	(77±10.9) (-9.27)	(64.40±9.69) (-24.56)	0.000*
200min	(78±9.22) (-8.09)	(62.73±8.21) (-26.52)	0.000*
210min	(82.61±9.76) (-2.66)	(63.23±8.20) (-25.93)	0.000*
220min	(83.5±11) (-2.20)	(63.60±8.06) (-25.50)	0.000*
230min	(84.5±13.2) (-0.44)	(63.90±7.54) (-25.15)	0.014*
240min	(79±0.00) (-6.92)	(64.40±7.66) (-24.56)	-
250min	-	(67.10±8.04) (-21.40)	-
260min	-	(68.33±7.26) (-19.96)	-
270min	-	(68.97±8.34) (-19.21)	-
280min	-	(70.26±9.01) (-17.77)	-
290min	-	(68.9±9.62) (-19.29)	-
300min	-	(70.13±9.3) (-17.85)	-
310min	-	(61±1.73) (-28.55)	-
320min	-	(58±0) (-32.06)	-
Values are Mean ± S.D. P-Value < 0.05 – Statistically Significant* P-Value > 0.05 – Statistically Not Significant (ns)			

The SBP, DBP and RR were comparable and no patients developed hypotension or respiratory depression in both groups (Table 3-4).

**Table 3:** Comparison of Changes in Systolic Blood Pressure

Time	Group C (Mean ± S.D.)	Group D (Mean ± S.D.)	P-Value (2 Sample t-Test)
Pre-operative	(127.63±15.33)	(130.27±12.89)	0.474 (ns)
1min	(122.67± 26.68) (-3.89)	(127.80±12.32 ) (-1.90)	0.344 (ns)
3min	(122.23±14.90 ) (-4.23)	(125.40±15.71 ) (-3.74)	0.426 (ns)
5min	(118.47±13.87 ) (-7.18)	(155.43±182.09 ) (19.32)	0.277 (ns)
10min	(115.37±15.67 ) (-9.61)	(118.63±14.92 ) (-8.93)	0.412 (ns)
15min	(115.57±13.04 ) (-9.45)	(117.20±12.81 ) (-10.03)	0.626 (ns)
20min	(114.77±12.60 ) (-10.08)	(116.57±12.14 ) (-10.52)	0.575 (ns)
25min	(112.67±11.87 ) (-11.72)	(116.37±12.62) (-10.67)	0.247 (ns)
30min	(111.93±10.55 ) (-12.30)	(116.03±9.61 ) (-10.93)	0.121 (ns)
40min	(109.80±12.03 ) (-13.97)	(112±10.28 ) (-14.02)	0.499 (ns)
50min	(111.93±12.02 ) (-12.30)	(111.57±11.39 ) (-14.36)	0.904 (ns)
60min	(113.13±11.64 ) (-11.36)	(110±9.41 ) (-15.36)	0.257 (ns)
70min	(114.47±10.03 ) (-10.31)	(110.20±10.06 ) (-15.41)	0.105 (ns)
80min	(116.27±9.56 ) (-8.90)	(110.80±9.09 ) (-14.95)	0.217 (ns)
90min	(116.13±14.93 ) (-9.01)	(113.40±10.49 ) (-12.95)	0.416 (ns)
100min	(115.67±12.47 ) (-9.37)	(113.23±11.20 ) (-13.08)	0.430 (ns)
110min	(116.50±12.73 ) (-8.72)	(112.83±11.30 ) (-13.39)	0.243 (ns)
120min	(116.40±14.47 ) (-8.80)	(114.20±11.36 ) (-12.34)	0.515 (ns)
130min	(115.57±13.19 ) (-9.45)	(113.33±9.51 ) (-13)	0.455 (ns)
140min	(116.20±12.36 ) (-8.96)	(114.77±9.60 ) (-11.90)	0.618 (ns)
150min	(116.93±12.14 ) (-8.38)	(115.83±10.96 ) (-11.08)	0.714 (ns)
160min	(116.17±14.85 ) (-8.98)	(114.23±9.30 ) (-12.31)	0.549 (ns)
170min	(116.73±12.79 ) (-8.54)	(119.27±22 ) (-8.45)	0.588 (ns)
180min	(118.57±13.24 ) (-7.10)	(116.40±9.30 ) (-10.65)	0.467 (ns)
190min	(116.2±13.8) (-8.96)	(118.10±9.16) (-9.34)	0.546 (ns)
200min	(118.3±13.5) (-7.31)	(114.1±11) (-12.41)	0.203 (ns)
210min	(116.9±10.3) (-8.41)	(114.63±10.95) (-12.01)	0.467 (ns)
220min	(116±13.1) (-9.11)	(115.53±10.94) (-11.31)	0.921 (ns)
230min	(113±15.2) (-11.46)	(117±12.33) (-10.19)	0.569 (ns)
240min	-	(116.97±9.85) (-10.21)	-
250min	-	(117.90±10.85) (-9.50)	-
260min	-	(118.70±10.76) (-8.88)	-
270min	-	(119.67±11.84) (-8.14)	-
280min	-	(119.84±10.3) (-8.04)	-
290min	-	(120.27±11.25) (-7.68)	-
300min	-	(118.2±11.8) (-9.27)	-
310min	-	(118.67±4.6) (-8.90)	-
320min	-	(127±0) (-2.51)	-
Values are Mean ± S.D. P-Value < 0.05 – Statistically Significant* P-Value > 0.05 – Statistically Not Significant (ns)			

**Table 4:** Comparison of Changes in Diastolic Blood Pressure

Time	Group C (Mean ± S.D.)	Group D (Mean ± S.D.)	P-Value (2 Sample t-Test)
Pre-operative	(75.30±10.02)	(83.8±10.36)	0.002 (ns)
1min	(73.37±7.28) (-2.57)	(80.63±9.53) (-3.78)	0.002 (ns)
3min	(69.33±14.38) (-7.92)	(76.13±9.87) (-9.15)	0.038 (ns)
5min	(67.87±9.41) (-9.87)	(73.87±9.24) (-11.85)	0.016 (ns)
10min	(64.67±9.17) (-14.12)	(70.37±10.26) (-16.03)	0.027 (ns)
15min	(65.97±8.36) (-12.39)	(69.13±10.68) (-17.50)	0.206 (ns)
20min	(66.33±9.89) (-11.91)	(72.50±11.42) (-13.48)	0.219 (ns)
25min	(65.5±8.54) (-13.01)	(72±12.05) (-14.08)	0.119 (ns)
30min	(66.33±7.90) (-11.91)	(69.97±11.26) (-16.51)	0.154 (ns)
40min	(66.63±7.90) (-11.51)	(69.47±11.04) (-17.10)	0.292 (ns)
50min	(66.77±8.55) (-11.33)	(69.23±12.93) (-17.38)	0.388 (ns)
60min	(67.80±8.30) (-9.96)	(68.20±11.30) (-18.62)	0.876 (ns)
70min	(70.67±8.40) (-6.15)	(67.97±10.31) (-18.89)	0.271 (ns)
80min	(71.47±7.73) (-5.09)	(69.27±11.67) (-17.34)	0.393 (ns)
90min	(69.50±8.09) (-7.70)	(69.97±11.75) (-16.51)	0.859 (ns)
100min	(69.37±7.85) (-7.88)	(70.37±10.88) (-16.03)	0.685 (ns)
110min	(68.63±9.17) (-8.85)	(72.03±11.06) (-14.04)	0.200 (ns)
120min	(70.13±10.77) (-6.86)	(72.83±10.07) (-13.09)	0.320 (ns)
130min	(68.80±9.51) (-8.63)	(72.97±9.25) (-12.93)	0.091 (ns)
140min	(70.13±11.52) (-6.86)	(73.53±10.48) (-12.25)	0.237 (ns)
150min	(70.43±9.94) (-6.46)	(73.47±9.53) (-12.33)	0.233 (ns)
160min	(70.57±10.90) (-6.29)	(74.23±9.59) (-11.42)	0.172 (ns)
170min	(70.50±9.08) (-6.37)	(73.70±9.26) (-12.05)	0.182 (ns)
180min	(70.67±10.80) (-6.15)	(73±9.27) (-12.89)	0.373 (ns)
190min	(69.3±11.2) (-7.97)	(74.47±11.29) (-11.13)	0.087 (ns)
200min	(70.3±9.6) (-6.64)	(73.9±10.27) (-11.81)	0.185 (ns)
210min	(71.78±7.9) (-4.67)	(73.90±10.28) (-11.81)	0.427 (ns)
220min	(69.5±6.47) (-7.70)	(72.37±10.99) (-13.64)	0.326 (ns)
230min	(69.67±5.39) (-7.48)	(71.67±9.75) (-14.47)	0.493 (ns)
240min	-	(72.93±10.78) (-12.97)	-
250min	-	(72.97±9.80) (-12.97)	-
260min	-	(71.47±9.28) (-14.80)	-
270min	-	(72.40±8.95) (-13.60)	-
280min	-	(72.4±9.8) (-13.60)	-
290min	-	(73.18±9.7) (-12.67)	-
300min	-	(70.8±6.9) (-15.51)	-
310min	-	(80.00±7) (-4.53)	-
320min	-	(83±0) (-0.95)	-
Values are Mean ± S.D. P-Value < 0.05 – Statistically Significant* P-Value > 0.05 – Statistically Not Significant (ns)			

Time taken for highest sensory level (TTHSL) and for complete motor block (TTCMB) was comparable between group C and D (12.78 ±5.07 mins and12.22 ± 4.11 mins for TTHSL) and (3.11 ±2.76 mins and 3.00 ±00 mins for TTCMB). [Table 5]. Time taken for sensory regression to L1 (TTSRL1) in group C was 191.11 ± 17.64 min and 278.89 ± 14.53 min in group D, showing a highly significant difference (P-value: 0.000*). There was a 31.2 % increase in TTSRL1 in group D vs group C. (Table 5). Time taken for complete motor recovery (TTCMR) also showed significant difference between the two groups, with group C at 212.22 ± 16.41 min and group D at 296.67 ± 10 min, with a 28% increase in group D as compared to group C (P-value: 0.000*) (Table 5). Time of rescue analgesia in group C was 120 mins and in group D the mean time for rescue analgesia was 197.26 min. On comparing both the groups, they were statistically significant (P-value 0.000*) (Table 5).

**Table 5:** Characteristics of Spinal Block and Rescue Analgesia

Parameters	Group C	Group D	P value (2 Sample t-Test)
HSLA	T_5_(T_4_-T_8_)	T_5_(T_4_-T_8_)	-
TTHSL	12.78±5.07	12.22±4.11	0.774 (ns)
TTCMB	3.11±2.76	3.00±00	0.738 (ns)
TTSRL1	191.11±17.64	278.89±14.53	0.000*
TTCMR	212.22±16.41	296.67±10	0.000*
RAT	120±0	197±26.80	0.000*
Values are either Median (Range ) or Mean ± S.D HSLA – Highest sensory level attained. TTHSL – Time taken for highest sensory level. TTCMB – Time taken for complete motor blockade. TTSRL1 – Time taken for sensory regression to L1. TTCMR – Time taken for complete motor recovery. RAT – Rescue analgesia time. p-value < 0.05 – statistically significant* p-value>0.05 - statistically not significant (ns)			

**Figure 2:**
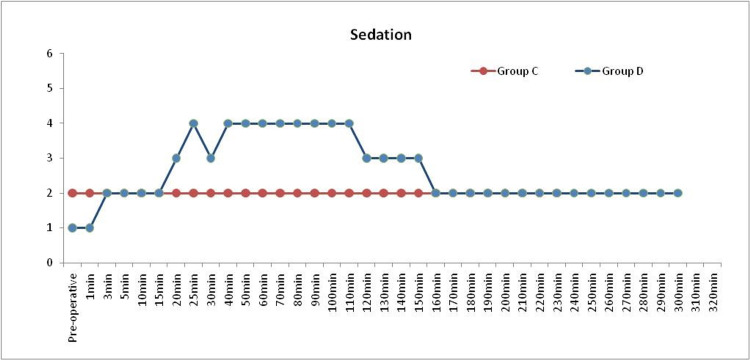
MEAN OF RAMSAY SEDATION SCORE IN BOTH GROUPS

Mean sedation scores in group C was 2 throughout the intraoperative and postoperative period. In group D, intraoperative Ramsay Sedation Score (RSS) of 3-4 was seen from 20mins to 110mins corresponding to the time of dexmedetomidine infusion. After stopping the infusion, the RSS reached a score of 2 postoperatively by 30 mins; this was clinically and statistically significant (P value <0.05*) (Table 6) (Figure 2).

**Table 6:** Comparison of Sedation levels between the two groups

Time	Median (Group C)	Median (Group D)	Mode (Group C)	Mode (Group D)	p-Value (Mann Whitney u test)
Pre-operative	2	1	2	1	0.011*
1min	2	1	2	1	0.02*
3min	2	2	2	2	0.158 (ns)
5min	2	2	2	2	0.967 (ns)
10min	2	2	2	2	-
15min	2	2	2	2	-
20min	2	3	2	3	-
25min	2	4	2	4	-
30min	2	4	2	3	-
40min	2	4	2	4	-
50min	2	4	2	4	-
60min	2	4	2	4	0.000*
70min	2	4	2	4	0.000*
80min	2	4	2	4	0.000*
90min	2	4	2	4	0.000*
100min	2	4	2	4	0.000*
110min	2	4	2	4	-
120min	2	3	2	3	-
130min	2	3	2	3	-
140min	2	3	2	3	0.000*
150min	2	3	2	3	0.000*
160min	2	2	2	2	0.000*
170min	2	2	2	2	-
180min	2	2	2	2	0.000*
190min	2	2	2	2	0.089 (ns)
200min	2	2	2	2	0.091 (ns)
210min	2	2	2	2	0.163 (ns)
220min	2	2	2	2	-
230min	2	2	2	2	-
240min	-	2	-	2	-
250min	-	2	-	2	-
260min	-	2	-	2	-
270min	-	2	-	2	-
280min	-	2	-	2	-
290min	-	2	-	2	-
300min	-	2	-	2	-
310min	-	2	-	-	-
320min	-	-	-	-	-
Values are in Median (range), Mode. P-Value < 0.05 – Statistically Significant* P-Value > 0.05 – Statistically Not Significant (ns)					

## Discussion

Subarachnoid block (SAB) with 0.5% hyperbaric bupivacaine is a widely used regional anesthetic technique, and is particularly advantageous for abdominal and lower limb surgeries lasting about 2-2.5 hours [5,6]. Several additives such as opioids and α agonists among others have been used with local anesthetics to prolong the duration of subarachnoid block [7]. Opioids have attained an integral role as a spinal anesthetic adjuvant, but their addition may lead to pruritus and respiratory depression [8]. Alpha 2 receptor agonists, like clonidine (α2R:α1R ratio of 200:1), are also being used [9]. A recent study suggested that an agonist with higher α2 receptor selectivity would show a more potent analgesic effect without causing hypotension like clonidine.

Dexmedetomidine is a selective α2-adrenoceptor agonist having analgesic and sedative effects and was primarily used for intravenous sedation [10]. The use of dexmedetomidine as a local anesthetic adjuvant has been increasingly reported to extend the duration of both motor and sensory blockade produced by single injection neuraxial [11-14] and peripheral [15-18] nerve blockade. Central mechanisms have been suggested to explain this action [19-21] and have proposed that routes of administration other than the intrathecal route may produce similar effects.

The mechanism by which intravenous dexmedetomidine prolongs the motor and sensory block of bupivacaine is by providing anesthetic and analgesic action through supraspinal action [22,23]. Single-dose intravenous injection and continuous infusion starting with a loading dose are the widely used methods to achieve its effects on anesthesia and analgesia during local or general anesthesia. We studied the effect of intravenous dexmedetomidine after a loading and maintenance dose on the quality of sensory and motor blockade.

Both the groups in our study showed similar onset of sensory and motor blockade. Previous studies done by Ahmed et al [24] compared three groups, group B received NS, group IV received intravenous dexmedetomidine 5 mins after SAB, group IT received intrathecal dexmedetomidine. They found that the time to reach Modified Bromage 3 motor block was significantly shorter in both IV and IT groups than in group B with no statistically significant difference between each other. Harsoor et al [25] who studied the effect of supplementation of low-dose intravenous dexmedetomidine (bolus 0.5 ug/kg then infusion 0.5 ug/kg/h before SAB) on characteristics of bupivacaine spinal anesthesia reported that administration of intravenous dexmedetomidine prolonged the duration of motor block and accelerated the onset of sensory block, but this accelerated motor and sensory block can be attributed to the time of infusion of dexmedetomidine after SAB.

The time taken for sensory regression to L1 and complete motor recovery in our study was prolonged in the group receiving dexmedetomidine. Findings similar to our study were reported by Jung et al [26] who studied the effects of single-dose intravenous dexmedetomidine on hyperbaric bupivacaine spinal anesthesia. They found that the two-dermatome sensory regression time was significantly increased with intravenous dexmedetomidine at doses of 0.25 and 0.5 ug/kg administered 5 mins after SAB. Lugo et al [27] noted prolongation of sensory block and duration of analgesia without significant effect on the motor block while using 1 ug/kg bolus followed by 0.5 ug/kg/h infusion of dexmedetomidine. Al-Mustafa et al [1] used a loading dose of 1 ug/kg dexmedetomidine over 10 min and a maintenance dose of 0.5 ug/kg/hr and observed similar findings in their study, and there was a prolongation of motor blockade while using a higher intravenous dose of dexmedetomidine.

The intraoperative Ramsay Sedation Scale (RSS) score was significantly higher in the dexmedetomidine group in our study and none of our patients had RSS greater than 4 at any point of observation. Dexmedetomidine produces sedation by its central effect and seems to be dose-dependent. Most of our patients receiving dexmedetomidine were sedated, but easily arousable. Mustafa et al [1] and Hong et al [28] in their study noted excessive sedation in 3 out of 25 and 2 out of 26 patients respectively. Kaya et al [29] also made a similar observation regarding sedation in their study. While comparing intravenous dexmedetomidine and midazolam, they found that the maximum Ramsay sedation score was greater in the dexmedetomidine and midazolam groups than in the saline group (P < 0.001). No Respiratory depression was seen in patients who received intravenous dexmedetomidine in our study.

Another significant finding in our study was the increased time taken for rescue analgesia requirement during the early postoperative period in patients who received dexmedetomidine. Similarly, Abdallah et al [30] showed that the time to first analgesic request was increased by at least 53% in the dexmedetomidine group and this was similar to our study which showed a 39% increase in the time taken to request analgesia.

In our study, 50% of patients showed bradycardia in group D, out of which only 10% of patients had HR ≤50/min which was transient and was treated with injection glycopyrrolate. The hemodynamic response following dexmedetomidine infusion depends upon the dose and speed of infusion. It has been noted that bradycardia is a prominent side effect, with incidence varying from 30% to 40% sometimes requiring treatment with atropine, following use of a bolus dose of 1 ug/kg and infusion greater than 0.4 ug/kg/h [3]. Our study showed no differences in blood pressure between the groups similar to that described by Abdallah et al [30] study. 

Our study was limited by our usage of a fixed dose of 3.5 ml 0.5% hyperbaric bupivacaine without taking into account, variations in height and weight of patients, which might be a confounding factor. Also, we did not compare the effects of dexmedetomidine with any other adjuvants to spinal anesthesia, and so further studies may be conducted in this regard.

## Conclusions

Intravenous dexmedetomidine 1ug/kg as a loading dose over 10 mins followed by a maintenance dose of 0.5ug/kg 15 mins after SAB appears to prolong the duration of sensory and motor blockade with no effect on onset and level of sensory block and onset of motor block. It also appears to provide sedation intraoperatively and analgesia postoperatively while maintaining hemodynamic stability with no significant respiratory depression and side effects.
